# Using more than 801 296 small-molecule crystal structures to aid in protein structure refinement and analysis

**DOI:** 10.1107/S2059798316014352

**Published:** 2017-02-22

**Authors:** Jason C. Cole, Ilenia Giangreco, Colin R. Groom

**Affiliations:** a Cambridge Crystallographic Data Centre, 12 Union Road, Cambridge CB2 1EZ, England

**Keywords:** macromolecular crystallography, Cambridge Structural Database, scripting

## Abstract

The Cambridge Structural Database (CSD) is the worldwide resource for the dissemination of all published three-dimensional structures of small-molecule organic and metal–organic compounds. This paper briefly describes how this collection of crystal structures can be used en masse in the context of macromolecular crystallography. Examples highlight how the CSD and associated software aid protein–ligand complex validation, and show how the CSD could be further used in the generation of geometrical restraints for protein structure refinement.

## Abstract 2

A guide to how the Cambridge Structural Database can be used to aid macromolecular crystallography.

## 1. Introduction   

The Cambridge Structural Database (CSD; Groom *et al.*, 2016 ▸) is a carefully curated collection of more than 800 000 structures of organic and metal–organic compounds provided by the Cambridge Crystallographic Data Centre (CCDC). The CSD has proven to be an invaluable resource for chemistry since its creation in 1965, and is heavily used in pharmaceutical research and development as well as in academic research. It is to the physical sciences what the Protein Data Bank (PDB; Berman *et al.*, 2003 ▸) is to the life sciences, but the CSD is well used to further protein crystallographic methods. Indeed, the paper by Engh and Huber describing parametrizations for macromolecular refinement (Engh & Huber, 1991 ▸) opens with the sentence

Bond-length and bond-angle parameters are derived from a statistical survey of X-ray structures of small compounds from the Cambridge Structural Database.

Historically, the elucidation of protein structures was driven by interest in discovering biological mechanisms; consequently, the focus of crystallographic methods was often on the protein component of a crystal structure, and less effort was expended on any associated ligands, as these were often peripheral to the critical information that a protein structure could provide. In recent years, there has been significant growth of interest in structures in the PDB directed at structure-based drug design, where protein–ligand crystal structures can be used to provide guidance to chemists in optimizing binding of small molecules to drug targets. This has been accompanied by the development of superb software that makes macromolecular crystallography accessible to those without a strong chemical background. Consequently, there has been a drive to improve methods for handling small molecules bound within macromolecular structures.

Chemical crystallography and macromolecular crystallo­graphy can be of mutual interest and benefit. Small-molecule crystallography can be useful to provide plausible hypotheses for molecular conformation. Such mutual relationships have always existed between the crystallographic communities and carry on today. An early example (Watson *et al.*, 1993 ▸) of such synergy is the case of conformations of glucose analogues bound to glycogen phosphorylase. These analogues were initially modelled in classical chair conformations when bound to protein structures, until a small-molecule structure showed that in certain cases glucose analogues could occupy a skew-boat conformation. In a more recent example (Tatum *et al.*, 2013 ▸), the use of small-molecule crystallography provided accurate starting models for input to docking studies of binding to the mycobacterial mono-oxygenase EthA. The small-molecule structures generated also aided in interpretation of the likely conformational changes undergone by the ligands on binding. Such uses of small-molecule crystallography are invaluable and frequent but are often underappreciated.

The need for better handling of ligand structures has been highlighted historically (Liebeschuetz *et al.*, 2012 ▸) and consequently efforts have been made to improve the tools and practices adopted in this area (Adams *et al.*, 2016 ▸). The CSD has been used as part of validation protocols (Read *et al.*, 2011 ▸) and in the generation of dictionaries of restraints (Moriarty *et al.*, 2016 ▸; Vagin *et al.*, 2004 ▸); work has also been carried out to integrate *Mogul* (Bruno *et al.*, 2004 ▸) into *PHENIX* (Adams *et al.*, 2010 ▸) and *Coot* (Emsley *et al.*, 2010 ▸). CSD data are now used routinely in the generation of the wwPDB chemical component dictionary through the CRESTANO project (wwPDB News, 2015 ▸). Distance restraints for small-molecule and protein structures derived from CSD data are also used in *SHELXL* (Sheldrick, 2015 ▸).

## 2. Structural data and the *CSD-System*   

The CCDC provides a web service for researchers to access any individual structures (http://www.ccdc.cam.ac.uk), enabling them to view and download the enhanced data sets deposited with the CCDC. This service is freely available to anyone in the world. In addition, the *CSD-System* software contains a number of programs that are relevant to protein crystallography. This software is used daily by structural chemists in well over a thousand institutions, but is also of tremendous value to structural biologists.

The main hub of the *CSD-System* is the visualiser *Mercury* (Macrae *et al.*, 2008 ▸), which allows the exploration of crystallographic structures in three dimensions. From *Mercury* it is possible to perform various types of substructure query, either directly from a specific three-dimensional structure or by launching substructure searching using *ConQuest* (Allen, 2002 ▸). Both *Mercury* and *ConQuest* support the extraction of three-dimensional structural information from CSD structures.

The *CSD-System* contains two knowledge bases: databases of information derived from raw CSD data. *Mogul* (Bruno *et al.*, 2004 ▸) is a library of intramolecular geometries containing bond lengths and angles for acyclic and cyclic fragments, torsion angles for acyclic fragments, and ring fragments. Each parameter entry in *Mogul* is a collection of up to 10 000 observations taken from CSD structures. Each distribution is keyed on the nature of the chemical environment of the said parameter. The distributions of these values are highly correlated with energy calculations; for example, a particular value of a torsion angle observed in many structures represents a low-energy value (Allen *et al.*, 1996 ▸; Cruz-Cabeza *et al.*, 2012 ▸).

*IsoStar* (Bruno *et al.*, 1997 ▸) is a library of interaction information derived from the CSD, allowing the user to visualize the interaction preferences of small-molecule fragments. *SuperStar* extends the utility of this information to a protein environment (Boer *et al.*, 2001 ▸; Verdonk *et al.*, 1999 ▸).

The understanding of interactions and geometry from the CSD allows the generation of software such as *GOLD*, a protein ligand-docking system which is of tremendous value in interpreting, and challenging the interpretation of, ligands fitted to electron density.

## 3. Using the *CSD-System* to understand interactions   

**Figure 1:**
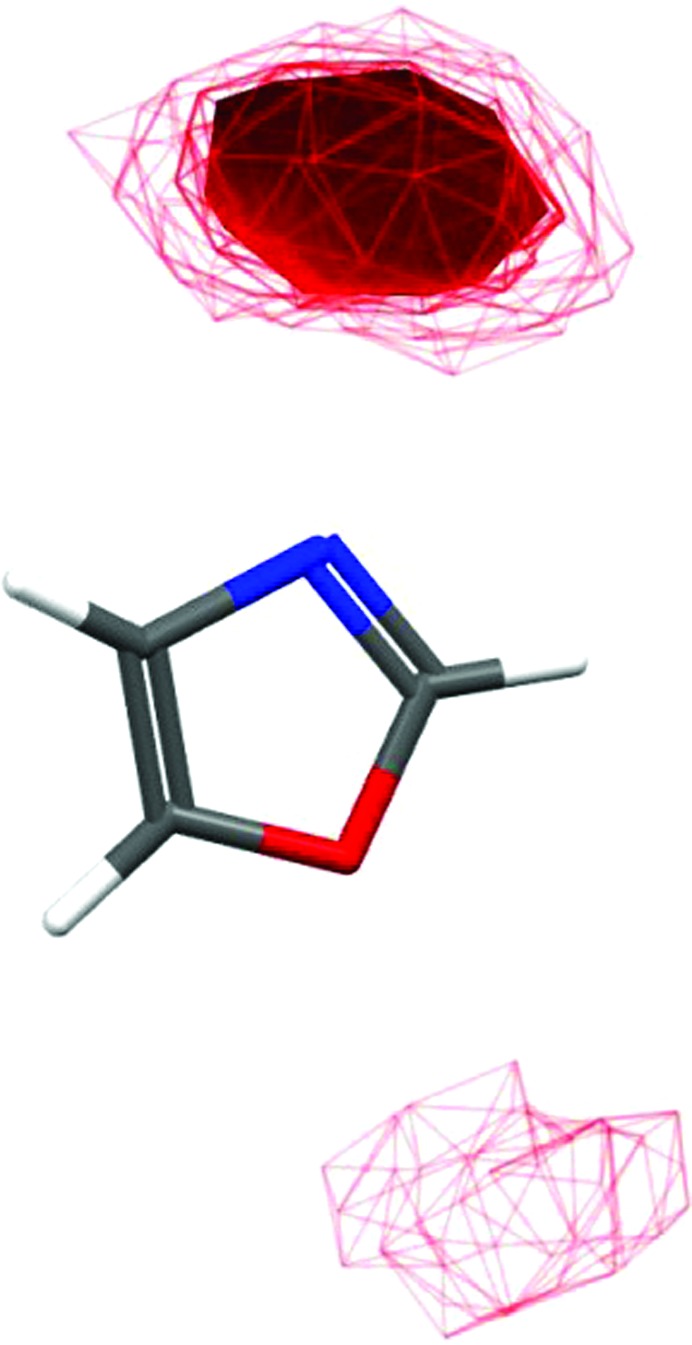
Contour plot showing the distribution of alcohol groups around oxazole rings. The more ‘solid’ the surface, the higher the likelihood of a group being in that position. None of the handful of alcohol groups near the O atom in small-molecule structures form hydrogen bonds. Such analyses can be performed using the interaction-map capabilities within *Mercury* or using the program *IsoStar*.

The individual molecules in a crystal lattice interact with their nearest neighbours. This allows one to generate statistical propensity values for the likelihood of any particular atom being in a certain position with respect to any other atom. Such propensities can be displayed graphically and compared with the observed interactions in a protein–ligand complex, either to validate the fit of a ligand or to guide chemical synthesis attempts to improve affinity. The range of questions that such tools can answer is incredible: for example, ‘I propose a fit whereby an F atom appears to act as a hydrogen-bond acceptor, is this likely?’, ‘my molecule contains a thiazole ring, would the nitrogen of an oxazole ring form similar interactions with the sulfur?’ and ‘my binding site contains a tryptophan residue, which functional groups are commonly seen to interact with these?’. To answer all such questions is far beyond the remit of this article, therefore we will restrict ourselves to one, fairly typical question: ‘my ligand contains an oxazole ring close to the hydroxyl group of a serine side chain. It is possible for me to fit this ligand such that either the nitrogen or the oxygen of the thiazole ring is closest to this serine, as I cannot distinguish between an N and an O atom in my electron-density maps. Which orientation is most likely?’ This question is trivial to answer and requires little computational or chemical expertise. A simple search of the CSD identifies over 8000 oxazole fragments, 52 of which hydrogen-bond to a hydroxyl group, all *via* the nitrogen (Fig. 1 ▸). One would, therefore need extremely strong evidence to position an oxazole ring in the alternative orientation.

**Figure 2:**
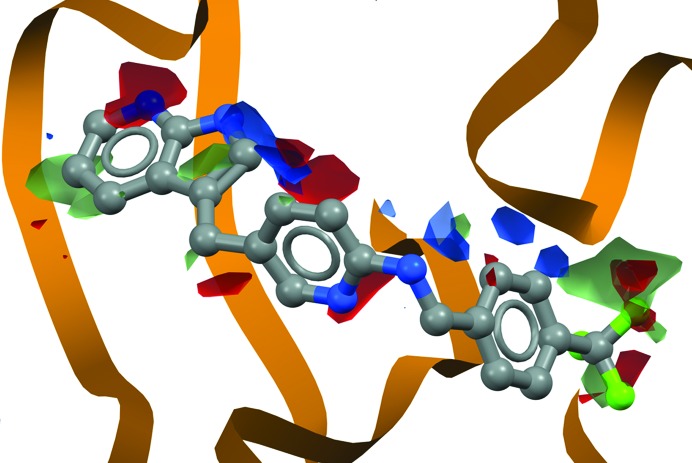
Analysis of protein–ligand interactions using CSD data for KIT kinase (PDB entry 4hvs; Zhang *et al.*, 2013 ▸). The bound inhibitor is superimposed on the interaction maps. The statistically most likely places one would find hydrogen-bond donors are shown in blue, hydrogen-bond acceptors in red and halogens in green. The maps show that these groups in the modelled ligand match the binding prediction well: it makes plausible interactions.

We can extend these approaches to the context of protein binding sites. Here, the same methodology is used to answer a question asked in a reverse manner: ‘Given the functional groups present in my binding site, where am I most likely to observe any particular group of my ligand?’ Fig. 2 ▸ illustrates the type of propensity maps one can generate. One would need convincing unbiased electron density to propose a fit of a ligand where its functional groups did not coincide with the probable positions determined from small-molecule crystal structure data. The dangers of such misinterpretation in macromolecular crystallography has recently been highlighted (Dauter *et al.*, 2014 ▸).

## 4. Using the *CSD-System* to understand molecular geometry   

**Figure 3:**
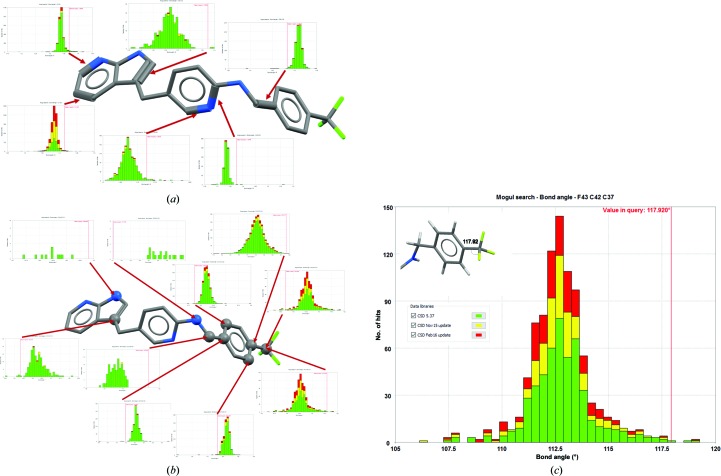
The distributions of geometrical parameters in the CSD compared with the ligand in PDB entry 4hvs. The thumbnail histograms represent observations in the CSD of bonds and valence angles between atoms identical to, and in similar environments to, the ligand. Red lines show the values observed in the ligand. The green bars of the histograms represent structures in the CSD as of August 2015, the yellow bars represent structures added between then and November 2015, and the red bars those added between November 2015 and February 2016. (*a*) Bond-length distributions. For the highlighted bonds, the values lie outside the range observed in small-molecule structures. (*b*) Bond valence angle distributions. For valence angles at the highlighted atoms, the values lie outside the range observed in small-molecule structures. Note that some CSD distributions are significantly more populated than others and also how the recent addition of many structures (*i.e.* red and yellow bars) better informs us of the expected valence angles of CF_3_ groups (*c*).

The CSD is also a library of molecular geometries and conformations. These are captured in the knowledge base *Mogul*. The most common application of *Mogul* in macromolecular crystallography is to check whether the geometry of a ligand modelled into electron density is plausible. Our indication of ‘plausibility’ is whether such geometry is common amongst the structures in the CSD. The coordinates of a modelled ligand can be loaded into the program *Mercury* and a geometry check performed. The ligand is automatically fragmented to match fragments for which distributions have been pre-calculated from relevant structures in the CSD. The approach taken considers both the chemical identity of a specific fragment and any nearby atoms in order to identify the most relevant distributions. A *Mogul* analysis for KIT kinase (PDB entry 4hvs; Zhang *et al.*, 2013 ▸) is shown in Fig. 3 ▸.

This structure was modelled into electron density calculated from virtually complete data collected to 1.9 Å resolution, and refined to an *R* value of 0.20 and a free *R* value of 0.227. It is a structure where the ligand is actually the focus of the study, by a company specializing in structure-based drug design. As we have seen from the *SuperStar* analysis above, the ligand has been modelled with the electron density such that its functional groups appear reasonably close to what one would expect from distributions calculated from small-molecule structural data, and one is able to gain a broad understanding of the key interactions of the ligand. The structure is thus reasonably representative of others in the literature, and this is a key factor to remember. This is why we have used this structure as an example, not to in any way criticize it, the science behind it, or the processing of it, but to demonstrate how the limited data available to a macromolecular crystallographer should be complemented by the use of small-molecule structures. Complementing macromolecular data in such a way mitigates against several difficulties that a protein crystallographer commonly faces: the need to use restraints, difficulties in distinguishing between C, N and O atoms, the inability to observe H atoms, difficulties in placing water molecules and multiple choices in how to interpret electron density.

Returning to our example, there are many small-molecule structures that provide information about every bond length, bond angle, torsion angle and ring system in this ligand. No element of this molecule represents unusual chemistry, so one might be surprised that all regions of the molecule show bond lengths and angles that are significantly different from those seen in small molecules. The azaindole ring is a key part, forming characteristic hydrogen bonds to the target kinase. There are over a hundred small-molecule crystal structures containing this precise ring system; however, three of the bonds and two of the valence angles in this ring have values that are unlikely, undoubtedly owing to the application of inappropriate restraints. The same is observed for the central linker of the molecule. The trifluoromethylphenylmethyl­amino tail does have bond lengths that fall within their range of expectation, but the same cannot be said of the valence angles, which in some cases differ by as much as 9° from those established by small-molecule crystallography. Any one of these bond lengths and angles would prevent the rest of the ligand fitting the electron density, so errors propagate throughout the structure; the fit to electron density has been achieved by distorting much of the molecule from the expected geometry based on prior observations, as seen in Fig. 3 ▸. Such geometrical analyses are trivial to perform, being generated in seconds by a few mouse clicks or a simple Python script, and should be a routine part of ligand fitting and analysis.

Although this ligand in PDB entry 4hvs is modelled in the correct place, the structure is of limited value in terms of understanding the principles of molecular geometry or recognition; it cannot, for example, be used to train scoring functions or evaluate molecular-docking programs. Furthermore, it could be misleading in a medicinal chemistry program, as the substitution vectors one would determine from this geometry would be misleading.

Interpretations such as this demonstrate the value of using the data in the CSD, and the *Mogul* tool in particular, to understand the geometry of a molecule *whilst* fitting to electron density and generating appropriate restraints. The latter can be achieved through using systems such as *grade* (Global Phasing), whereby restraints are generated from small-molecule structural data, complemented by calculations when necessary. This enables ligands to be refined with chemically sensible geometry at the outset and allows the structural biologist to generate the most plausible fit of the ligand to density.

## 5. Using the *CSD-System* in ligand fitting   

**Figure 4:**
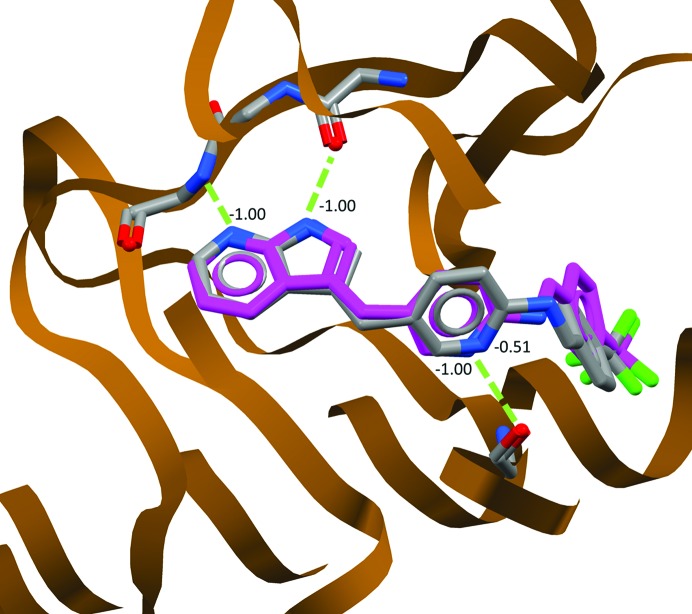
Docking of the 4hvs ligand using *GOLD* (shown in magenta), superimposed on the modelled ligand from the PDB structure (grey). The numbers represent the contribution of hydrogen-bonding groups to the docking score. Both N atoms of the azaindole ring are positioned well to make hydrogen bonds (green dashes), reflected in a ‘perfect’ score of −1.00. However, the N atom of the pyridine ring in the modelled crystal structure is rather poorly positioned to hydrogen-bond, reflected in its score of −0.51. The docking software positions the ligand slightly differently in the binding site and improves the geometry of the ligand, such that the atom is now in a perfect position to hydrogen-bond.

The sections above describe how one can use specific information derived from the CSD to understand and perhaps optimize the geometry and interactions of a bound ligand. This can be of enormous help in the interpretation of electron density; however, more automated approaches are possible. The protein–ligand docking program *GOLD*, which is part of the *CSD-Enterprise* system available to all academic researchers, combines an understanding of both molecular geometry and interactions to generate plausible docking modes for ligands in protein structures (Fig. 4 ▸). These docking modes provide ‘electron-density naïve’ views of how a ligand might bind to a protein, giving the crystallographer unbiased alternatives for consideration when interpreting electron density. Docking methods such as this rely on relatively simple atom–atom scoring functions, which balance summed inter­action scores against considerations of ligand geometry. Of particular help is the ability to visualize the individual atomic contributions to these scores. Atoms scoring poorly may be contributing little to the binding of a ligand, or may be indicative of a misinterpretation of the electron density. Again, one would need compelling electron density to propose a fit of a ligand where its functional groups did not coincide with where docking software suggested they were most likely to be. Of course, where this data exists, differences between docking predictions and experimental predictions provide a wealth of ideas for the synthetic chemist.

## 6. Using the *CSD-System* to understand protein structure   

As we have mentioned, Engh & Huber (1991 ▸) generated bond-length and bond-angle parameters for use in refinement of macromolecular crystal structures using information from the CSD. At the time of their work, the CSD contained around 100 000 entries; we now have over 800 000 structures available, allowing us to improve our restraints. As noted by Touw & Vriend (2010 ▸), the original Engh & Huber parameters overgeneralize, a known and inevitable consequence of the data available at the time. They illustrate this by showing how the τ angle at the α-carbon in a peptide chain is dependent on the broader environment of the peptide.

**Figure 5:**
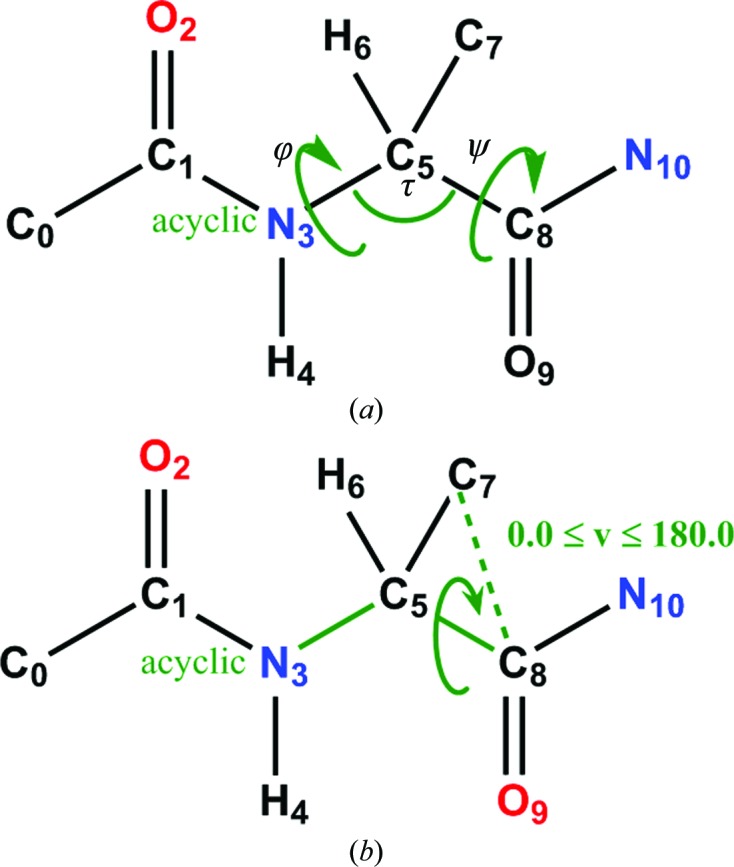
Query used to return relevant dipeptides from the CSD. (*a*) Key peptide query and returned data parameters. (*b*) An improper torsion angle used to constrain the search to l-amino-acid dipeptide fragments. To ensure that we only extract peptidic fragments from l-amino-acid systems, we constrain the results so that the improper torsion angle C7–C8–C5–N3 is in the range 0.0–180°.

Evidence of this is extremely clear in the CSD, which contains many peptidic structures, which are easily found using the query graphically represented in Fig. 5 ▸.

**Figure 6:**
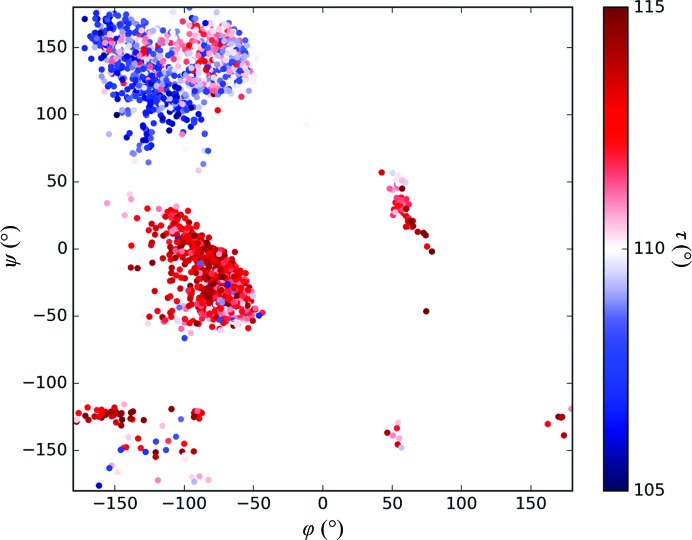
Ramachandran plot of CSD entries containing dipeptides. The plot is coloured from blue to red based on τ, the backbone valence angle subtended at the α-carbon. A small number of entries with extreme values of τ (predominantly in cyclized or metal-bound structures) were removed.

The values of φ, ψ and τ are easily extracted from a CSD search, so one can create a Ramachandran plot of ψ *versus* φ, coloured by the value of τ. As is observed in Fig. 6 ▸, small-molecule structures with ψ and φ values observed in helices tend towards larger values of τ, a fact recognized by a number of researchers (Moriarty *et al.*, 2014 ▸, 2016 ▸; Tronrud & Karplus, 2011 ▸). These changes in this angle allow particularly favourable hydrogen-bonding geometry (Wood *et al.*, 2009 ▸) to occur along α-helices, by allowing the carbonyl oxygen vector to twist out from the axis of the helix. This results in more linear hydrogen bonds with amide nitrogen groups one turn along a helix, a point recognized some time ago in the seminal work of Baker & Hubbard (1984 ▸).

The purpose of this example is to demonstrate that it is straightforward to extract not just ligand-relevant but also protein-relevant data from CSD searches. One obvious extension would be to automate the production of refinement restraints so that they are generated on-the-fly from only the most relevant high-resolution multi-peptide small-molecule structures (*i.e.* those with similar secondary structure, or the same or following residues) rather than from the amino-acid base units only.

## 7. Conclusions   

We generated our examples using both the graphical user interfaces provided within *CSD-Enterprise* and the *CSD-System* Python application programming interface (API; CCDC, 2015 ▸). This allows both script-based access to the functionality and provides a way to integrate this functionality into software commonly used by macromolecular crystallo­graphers.

The above examples highlight how the CSD could be, should be, must be and is used to great effect by macromolecular crystallographers, particularly those interested in protein–ligand binding. We hope that we have gone a little way in bringing back together the small-molecule and macromolecular approaches that are so synergistic.
