# Using more than 801 296 small-molecule crystal structures to aid in protein structure refinement and analysis. Addendum

**DOI:** 10.1107/S2059798317016904

**Published:** 2017-11-30

**Authors:** Jason C. Cole, Ilenia Giangreco, Colin R. Groom

**Affiliations:** a Cambridge Crystallographic Data Centre, 12 Union Road, Cambridge CB2 1EZ, England

**Keywords:** macromolecular crystallography, Cambridge Structural Database, scripting, addendum

## Abstract

An addendum to the *Introduction* of Cole *et al.* [(2017), *Acta Cryst.* D**73**, 234–239] is made to recognize the work of Bricogne, Smart and others in the development of methods to make use of Cambridge Structural Database data in protein structure solution.

## Abstract 2

An addendum to the article by Cole *et al.* [(2017), *Acta Cryst.* D**73**, 234–239].

In the *Introduction* to Cole *et al.* (2017 ▸) a short resume of example areas where *Mogul* (Bruno *et al.*, 2004 ▸) has been used was given. Two contributions in this area (*BUSTER* and *GRADE*; Smart *et al.*, 2011 ▸) were the pioneering works where *Mogul* was applied to protein crystallography, and so should also have been recognised.
